# Practice of martial arts and bone mineral density in adolescents of both sexes

**DOI:** 10.1016/j.rppede.2015.09.003

**Published:** 2016

**Authors:** Igor Hideki Ito, Alessandra Madia Mantovani, Ricardo Ribeiro Agostinete, Paulo Costa, Edner Fernando Zanuto, Diego Giulliano Destro Christofaro, Luis Pedro Ribeiro, Rômulo Araújo Fernandes

**Affiliations:** a Instituto de Biociências, Universidade Estadual Paulista "Júlio de Mesquita Filho" (Unesp), Rio Claro, SP, Brazil; b Universidade Estadual Paulista "Júlio de Mesquita Filho" (Unesp), Presidente Prudente, SP, Brazil; c Universidade do Algarve, Faro, Portugal

**Keywords:** Martial arts, Bone mineral density, Adolescents

## Abstract

**Objective:**

The purpose of this study was to analyze the relationship between martial arts practice (judo, karate and kung-fu) and bone mineral density in adolescents.

**Methods:**

The study was composed of 138 (48 martial arts practitioners and 90 non-practitioners) adolescents of both sexes, with an average age of 12.6 years. Bone mineral density was measured using Dual-Energy X-ray Absorptiometry in arms, legs, spine, trunk, pelvis and total. Weekly training load and previous time of engagement in the sport modality were reported by the coach. Partial correlation tested the association between weekly training load and bone mineral density, controlled by sex, chronological age, previous practice and somatic maturation. Analysis of covariance was used to compare bone mineral density values according to control and martial arts groups, controlled by sex, chronological age, previous practice and somatic maturation. Significant relationships between bone mineral density and muscle mass were inserted into a multivariate model and the slopes of the models were compared using the Student *t* test (control versus martial art).

**Results:**

Adolescents engaged in judo practice presented higher values of bone mineral density than the control individuals (*p*-value=0.042; Medium Effect size [Eta-squared=0.063]), while the relationship between quantity of weekly training and bone mineral density was significant among adolescents engaged in judo (arms [*r*=0.308] and legs [*r*=0.223]) and kung-fu (arms [*r*=0.248] and spine [*r*=0.228]).

**Conclusions:**

Different modalities of martial arts are related to higher bone mineral density in different body regions among adolescents.

## Resumo

**Objetivo:**

Analisar a relação entre a prática de artes marciais (judô, karatê e kung-fu) e a densidade mineral óssea em adolescentes.

**Métodos:**

O estudo foi composto por 138 adolescentes (48 praticantes de artes marciais e 90 não praticantes) de ambos os sexos, com média de 12,6 anos. A densidade mineral óssea foi medida com absortometria radiológica de dupla energia em braços, pernas, coluna, tronco, pelve e total. A carga de treinamento semanal e o tempo anterior de envolvimento na modalidade esportiva foram relatados pelo treinador. A correlação parcial testou a associação entre a carga semanal de treinamento e a densidade mineral óssea, controlada para sexo, idade cronológica, prática anterior e maturação somática. A análise de covariância foi usada para comparar os valores de densidade mineral óssea de acordo com os grupos controle e de artes marciais, controlados para sexo, idade cronológica, prática anterior e maturação somática. Associações significativas entre a densidade mineral óssea e a massa muscular foram inseridas em um modelo multivariado e as inclinações dos modelos foram comparadas com o teste *t* de Student (controle *versus* arte marcial).

**Resultados:**

Os adolescentes envolvidos na prática de judô apresentaram valores maiores de densidade mineral óssea do que os do grupo controle (*p*=0,042; tamanho de efeito médio [eta-quadrado=0,063]), enquanto a relação entre a quantidade de treinos semanais e a densidade mineral óssea foi significativa entre os adolescentes praticantes de judô (braços [r=0,308] e pernas [r=0,223]) e kung-fu (braços [r=0,248] e coluna [r=0,228]).

**Conclusões:**

Diferentes modalidades de artes marciais estão relacionadas com maior densidade mineral óssea em diferentes regiões do corpo em adolescentes.

## Introduction

During adulthood, bone health is affected by physical inactivity and aging, since both affect bone structure and can lead to osteoporosis and fractures.^1^ Therefore, physical exercise is widely advocated for the prevention of fractures and osteoporosis through increases in bone mineral density (BMD) and reduction in age-related bone loss.^2^ In adulthood, physical exercise produces low increments in bone mass^2^ and, thus, childhood and adolescence appear to be the most significant periods in which to improve BMD^2^^,^ 3 and, hence, prevent outcomes such as osteoporosis in adulthood.

During childhood and adolescence growth hormone contributes to bone mass gain and the concentration of this hormone in the blood is increased by physical exercise.^4^ Furthermore, the strength and geometry of bone are substantially affected by the higher continuous muscle contractions observed in sport activities.^5^ Therefore, the practice of physical activity is recommended and some studies have reported improvement in BMD in many modalities^6^^-^ 8 such as soccer,^9^ volleyball^10^ and badminton;^11^ but this event is less reported in martial arts.^12^

Martial arts include high-magnitude forces through muscle pulling on the bone, ground reaction forces intensified by the absence of footwear to attenuate impact shocks and high-impact loading of the skeleton due to repeated falls on the ground.^13^ The American College of Sports Medicine^14^ recognizes the beneficial effect of sports practice on bone mass gain during human growth, but the same statement identifies that, although martial arts have aspects related to BMD gain, the findings are mainly based on elite athletes,^6^ and it is not clear if this relationship occurs in children and adolescents.^15^ Moreover, the absence of control by important variables related to bone mass gain during childhood and adolescence (fat free mass [FFM] and biological maturation) constitutes a limitation in studies analyzing the relationship between sport practice and BMD in Pediatric populations.^16^

Therefore, the aims of this study were (i) to verify the relationship between widely performed martial arts (judo, karate and kung-fu) and BMD in adolescents, as well as (ii) to identify whether this relationship is independent of biological maturation and FFM. We hypothesized that martial arts practice would be related to higher BMD in adolescents of both sexes.

## Method

This cross-sectional study was composed of 138 adolescents of both sexes (ages ranging from 11 to 14 years) and was carried out in the "Laboratory for the InVestigation of Exercise" (LIVE) of the Department of Physical Education, Universidade Estadual Paulista "Júlio de Mesquita Filho" (UNESP), in the city of Presidente Prudente, Brazil. The cohort study "Practice of different sport modalities and bone mass gain in adolescents: cohort of 9 months" was conducted during 2013 and 2014 and the presented data forms part of the baseline measures.

Sample size estimation was performed using an equation based on analysis of variance (ANOVA), which took into account a minimum difference for BMD of 0.255 (g/cm^2^)^13^ between the control and martial arts groups, a standard deviation of 0.180g/cm^2^, four independent groups (Control, Karate, Kung-fu and Judo), a power of 80% and alpha of 5%. Final sample size was estimated as a minimum of 12 adolescents per group and, therefore, at least 48 adolescents were required. For adolescents engaged in martial arts, the following criteria were included: (i) aged between 11 and 17; (ii) a minimum of 03 months of previous involvement in the current martial art and (iii) the coach's permission. Adolescents of both sexes, engaged in martial arts (regional level), were recruited from sport clubs in different regions of the city as follows: Karate (9 girls and 5 boys [*Shotokan* style]), Judo (8 girls and 10 boys) and Kung-fu (1 girl and 15 boys [*sanda* style]). The control group was composed of adolescents from five schools located in different regions of the city. In these five schools, all students aged between 11 and 17 were invited to participate and after confirming that they met the inclusion criteria, ([i] aged between 11 and 17 and [ii] not engaged in regular physical activity/sport outside school), the control group was composed of 36 girls and 54 boys.

The parents or guardians signed a written consent form and the study had been previously approved by the Ethical Board of the Sao Paulo State University Campus of Presidente Prudente, Brazil.

Body weight was measured using a digital scale (Filizzola, Sao Paulo, Brazil, to the nearest 0.1kg) and height was measured with a wall-mounted stadiometer (to the nearest 0.1cm) with a maximum length of 200cm. All anthropometric measurements were performed following previously published standard methods.^17^ The leg length and sitting-height were also assessed using standardized techniques.^17^ These measurements were used to calculate the maturity offset, which denotes the time (years) from/to peak of height velocity (PHV),^18^ an important maturational event. PHV is an indicator of somatic maturity (linear growth) and reflects the age of maximum rate of growth in stature during adolescence.

Weekly training load was measured by means of a questionnaire filled in by the coach, in which the following questions about each athlete were asked: (i) the number of days per week and (ii) number of hours per day spent training. Weekly training load was calculated using the equation: number of days×hours per day (expressed in minutes/week). Time of previous engagement in the martial art was also reported by the coach (in months), as well as the use of any supplementation (weight loss or muscle mass gain).

The bone mineral density (in g/cm^2^) in different body regions ([i] whole body BMD, [ii] BMD of the lower limbs, [iii] BMD of the upper limbs, [iv] BMD of the spine region, [v] BMD of the pelvis and [vi] BMD of the trunk) was analyzed using the Dual-Energy X-ray Absorptiometry (DEXA) technique. The device used was the Lunar model - DPX-NT (General Electric [GE]). The radiation dose that each participant received was less than 0.05mrem, in other words, equivalent to 50 times less than a conventional X-ray examination.^19^ All participants wore light clothing and were barefoot (no metal belongings on the body), and were placed on the equipment in the supine position, remaining motionless throughout the examination, approximately 15 min. The results were calculated by means of specific software provided by the manufacturer. Moreover, measures of body fatness (as a percentage) and FFM (in kg) were also provided by the DXA software.

Chronological age, sex (male or female), FFM and somatic maturation were treated as potential confounders in this study and, thus, were used to adjust the multivariate models. Chronological age (in a decimal scale) was estimated by the difference between birth date and assessment date.

The normality of data was analyzed using the Komogorov-Smirnov test (weekly training load presented non-parametric distribution and, thus, was log transformed). Descriptive statistics consisted of mean and standard-deviation values. Partial correlation analyzed the relationship between weekly training load and BMD controlled by sex, chronological age, time of previous practice and somatic maturation. Analysis of covariance (ANCOVA) was used to compare BMD values relating to the control and martial arts groups, controlled by sex, chronological age, time of previous practice and somatic maturation (the Bonferroni post hoc was used when necessary). Measures of effect size were provided through values of Eta-squared. Finally, significant relationships between BMD and FFM were inserted into a multivariate model (adjusted simultaneously by age, sex and somatic maturation) and the slopes of the models were compared using the Student *t* test (control versus martial art). All statistical procedures were performed using the software BioEstat (version 5.0 [Mamirauá Institute, Tefé - Brazil]) and the significance level (*p*-value) was set at *p*<0.05.

## Results

The sample of the present study was composed of 137 adolescents (47 engaged and 90 not engaged in martial arts). Significant differences between groups were observed for the variables of age (*p*-value=0.020), body weight (*p*-value=0.026) and somatic maturation (*p*-value=0.012). Adolescents engaged in martial arts presented higher BMD in their arms, legs, trunk, spine and total BMD (Table 1).

**Table 1:** General characteristics of the adolescents stratified by practice of martial arts (n=137).

Variables	Control group (n=90)	Martial arts group (n=47)	*p*-value
	Mean (SD)	Mean (SD)	
Age (years)	11.93 (0.90)	12.36 (1.01)	0.020
Weight (kg)	49.50 (11.36)	54.78 (15.52)	0.026
Stature (cm)	155.67 (7.73)	158.43 (11.31)	0.165
% body fatness	27.62 (12.43)	27.59 (12.56)	0.808
Fat free mass (kg)	32.43 (5.47)	35.35 (9.70)	0.065
Maturity offset^a^	-2.67 (0.72)	-2.26 (0.94)	0.012

*Bone mineral density g/cm* ^2^			
Arms	0.68 (0.06)	0.76 (0.19)	0.007
Legs	1.08 (0.10)	1.18 (0.23)	0.015
Trunk	0.83 (0.07)	0.88 (0.09)	0.002
Pelvis	1.11 (0.97)	1.07 (0.13)	0.790
Spine	0.89 (0.11)	0.95 (0.12)	0.002
Whole body	1.01 (0.07)	1.04 (0.09)	0.010

SD, Standard deviation.Fisher's exact test with *p*-value=0.020.

a Age at peak height velocity.

The ANCOVA identified that only adolescents engaged in judo practice presented higher values of BMD in the arms than the control group (Eta-squared=0.063; medium effect size) after controlling by sex, age, FFM and somatic maturation (Table 2).

**Table 2:** Analysis of covariance estimated means of bone mineral density in adolescents according to control and martial arts (n=137).

Variables	Control (n=90)	Judo (n=17)	Karate (n=14)	Kung-fu (n=16)		ANCOVA		
	Mean (SE)					*p* -value	Eta-squared	Effect size
Arms (g/cm^2^)	0.695 (0.01)	0.771 (0.02)^a^	0.695 (0.03)	0.735 (0.03)		0.042	0.063	Medium
Legs (g/cm^2^)	1.105 (0.01)	1.173 (0.03)	1.139 (0.04)	1.150 (0.04)		0.264	0.031	Small
Trunk (g/cm^2^)	0.845 (0.01)	0.841 (0.01)	0.863 (0.02)	0.876 (0.01)		0.243	0.032	Small
Pelvis (g/cm^2^)	1.122 (0.08)	1.028 (0.20)	1.098 (0.24)	1.089 (0.23)		0.979	0.001	Trivial
Spine (g/cm^2^)	0.904 (0.01)	0.897 (0.02)	0.944 (0.02)	0.963 (0.02)		0.083	0.051	Small
Whole body (g/cm^2^)	1.021 (0.01)	1.009 (0.01)	1.033 (0.02)	1.049 (0.02)		0.292	0.029	Small

ANCOVA, analysis if covariance controlled by sex, age, fat free mass and somatic maturation; SE, Standard error.

a *p*-value<0.05 compared to the control group.

Even after controlling for potential confounders, there were significant relationships between FFM and BMD in the control group (arms, legs, trunk, spine and whole body), Judo group (trunk, pelvis and spine) and Karate group (legs, trunk, pelvis, spine and whole body) (Table 3). There was a stronger relationship between FFM and whole body BMD in the control and Karate groups than the Kung-fu group. Similar patterns were also observed for spine, trunk and leg BMD.

**Table 3:** Slope comparisons of the relationship between fat free mass and bone mineral density in adolescents of different martial arts (n=138).

Bone mineral density (g/cm^2^)	Control (n=90)	Judo (n=17)	Karate (n=14)	Kung-fu (n=16)
	FFM (kg)	FFM (kg)	FFM (kg)	FFM (kg)
	β (β 95%CI)^a^	β (β 95%CI)^a^	β (β 95%CI)^a^	β (β 95%CI)^a^
Arms (g/cm^2^)	**0.006 (0.004 to 0.009)**	0.005 (-0.069 to 0.080)	0.010 (-0.001 to 0.020)	0.004 (-0.002 to 0.010)
Versus control	-	*p*-value=0.976	*p*-value=0.435	*p*-value=0.528
Legs (g/cm^2^)	**0.013 (0.010 to 0.017)**	0.010 (-0.085 to 0.105)	**0.026 (0.011 to 0.042)**	0.003 (-0.003 to 0.009)
Versus control	-	*p*-value=0.945	*p*-value=0.077	*p*-value=0.006
Trunk (g/cm^2^)	**0.010 (0.007 to 0.012)**	**0.023 (0.006 to 0.040)**	**0.017 (0.005 to 0.028)**	0.001 (-0.003 to 0.005)
Versus control	-	*p*-value=0.110	*p*-value=0.173	*p*-value=0.001
Pelvis (g/cm^2^)	0.022 (-0.026 to 0.070)	**0.035 (0.007 to 0.064)**	**0.024 (0.007 to 0.040)**	0.001 (-0.006 to 0.005)
Versus control	-	*p*-value=0.635	*p*-value=0.936	*p*-value=0.385
Spine (g/cm^2^)	**0.012 (0.008 to 0.015)**	**0.031 (0.010 to 0.052)**	**0.026 (0.007 to 0.045)**	-0.001 (-0.008 to 0.007)
Versus control	-	p-value=0.065	*p*-value=0.132	*p*-value=0.002
Whole body (g/cm^2^)	**0.007 (0.005 to 0.010)**	0.016 (-0.001 to 0.032)	**0.017 (0.005 to 0.029)**	0.002 (-0.003 to 0.008)
Versus control	-	*p*-value=0.206	*p*-value=0.053	*p*-value=0.027

FFM, fat free mass.

a Model adjusted by sex, age and somatic maturation. Bold indicates *p*-value significant and positive correlation between fat free mass and bone mineral density.

Weekly training loads were 6.38±5.43hours/week for Judo, 10.46±2.78hours/week for Karate and 3.15±1.20hours/week for Kung-fu (*p*-value<0.001 among all martial arts). Even after controlling for potential confounders, BMD in the legs and arms was related to weekly training load in the Judo group. Similar relationships were identified in the Kung-fu group for arms and spine (Table 4). There were no significant relationships for the Karate group.

**Table 4:** Partial correlation between bone mineral density and weekly training load in adolescents of different martial arts (n=138).

Bone mineral density (g/cm^2^)	Control versus Judo(n=108)	Control versus Karate(n=104)	Control versus Kung-Fu(n=106)
	*r*(*r*95%CI)	*r*(*r*95%CI)	*r*(*r*95%CI)
Arms	**0.308 (0.126 to 0.470)**	0.018 (-0.175 to 0.210)	**0.248 (0.060 to 0.419)**
Legs	**0.223 (0.036 to 0.395)**	0.144 (-0.050 to 0.328)	0.185 (-0.006 to 0.363)
Trunk	0.012 (-0.177 to 0.201)	0.124 (-0.070 to 0.309)	0.171 (-0.020 to 0.350)
Pelvis	-0.018 (-0.206 to 0.172)	-0.008 (-0.200 to 0.185)	-0.013 (-0.203 to 0.178)
Spine	-0.009 (-0.198 to 0.180)	0.165 (-0.028 to 0.347)	**0.228 (0.039 to 0.401)**
Whole body	-0.071 (-0.257 to 0.120)	0.065 (-0.129 to 0.254)	0.149 (-0.043 to 0.330)

Model adjusted by sex, age, somatic maturation and fat free mass. Bold indicates a positive correlation between BMW and weekly training load.

## Discussion

The findings of the present study identified that adolescents engaged in martial arts presented higher BMD in different body regions; however BMD values were similar among the martial arts tested. Moreover, the relationship between FFM and BMD occurred at different magnitudes in the control group and different martial arts groups analyzed.

In the present study, the weekly training load presented a significant relationship with BMD, principally for the arms and legs. Regarding the upper limbs, Kung-fu and Judo presented a significant relationship between weekly training load and BMD when compared to the control group. The upper limbs are widely used in Judo practice and thus our findings were expected (higher values than the control group and a significant relationship with weekly training load). On the other hand, our findings also suggest that this specific Kung-fu fighting style (called *sanda*) has a significant impact on training and fighting, as it encompasses a large number of techniques with physical impact to the upper limbs, such as a wide variety of punches.^20^ Karate presented similar values to the control group, perhaps because Karate *shotokan* (the style studied) is considered a form of semi contact (during competitions, the physical contact is prohibited) and, thus, physical contact is not common in the training of this modality.

In this sample, no significant differences were found in the pelvis BMD of practitioners and non-practitioners, suggesting that these methods of combat do not include mechanisms for jumping or generating impact in the hip region in the way that volleyball and artistic gymnastics do.^21^ In highly trained athletes (between 18 and 25 years) karate and judo practice increases BMD more than the practice of water polo.^6^ In addition, judo and taekwondo practitioners presented higher BMD than runners.^22^ However, in the aforementioned studies,^6^^,^ 22 pelvis BMD was not assessed separately. Therefore, caution is necessary when generalizing these results.

Muscle mass and biological maturation have been pointed out as important determinants of the bone mass gain and geometry related to sport practice,^23^^,^ 24 predominantly because both variables are strongly related to each other during childhood and adolescence. On the other hand, the absence of muscle mass and biological maturation in studies analyzing BMD values and sport practice in adolescents constitutes a relevant limitation in the specialized scientific literature.^16^ In our study, sport practice affected BMD values independent of the somatic maturation status or values of FFM, which agrees with Ferry,^25^ who also identified that 8 months of soccer practice affected bone geometry even in post-pubertal adolescents. Apparently, the pathways linking sport practice (in this case, martial arts) and bone health during adolescence are probably boosted by biological maturation events, but these pathways seem to be independent of biological maturation.

Although some authors have found a positive effect of other sport modalities on BMD,^13^^,^ 23 ^,^ 24 ^,^ 26 there is no consensus about the ideal training load for young athletes. The guideline of the American College of Sports Medicine^14^ indicates that at least 120min/week are necessary to improve bone health. However, in an additional analysis, the cutoff proposed by the American College of Sports Medicine was not related to higher BMD in the analyzed sample (data not shown). Therefore, the most profitable combination between exercise intensity and quantity for targeting BMD gain in adolescents is still unclear and further research is necessary.

It is important to point out some limitations of this study; the cross-sectional design in which there is an absence of causality should be considered. Moreover, the absence of nutritional factors (e.g. calcium and vitamin D) and exposure to sunlight should be considered in future studies. All bone variables analyzed in the present study are strongly affected by biological maturation, which is commonly represented by the Tanner method (sexual maturation). In the present study PHV was adopted as a maturation marker, as PHV is a non-invasive method, which discriminates maturational events (hormonal patterns) as effectively as the Tanner method in both sexes.^27^ Finally, DXA does not provide measures of bone geometry, which could be useful in studies analyzing this issue.^25^

In conclusion, despite the limited understanding of the effects of martial arts on children and adolescents, a positive relationship between BMD and practice of martial arts was identified, although this relationship seems to be dependent on the martial art analyzed.
